# Supplementary figures and images for: Antipyretic Mechanism of Bai Hu Tang on LPS-Induced Fever in Rat: A Network Pharmacology and Metabolomics Analysis
Source: Pharmaceuticals (Basel). 2025 Apr 23;18(5):610. doi: 10.3390/ph18050610 (PMC12114418; doi:10.3390/ph18050610)

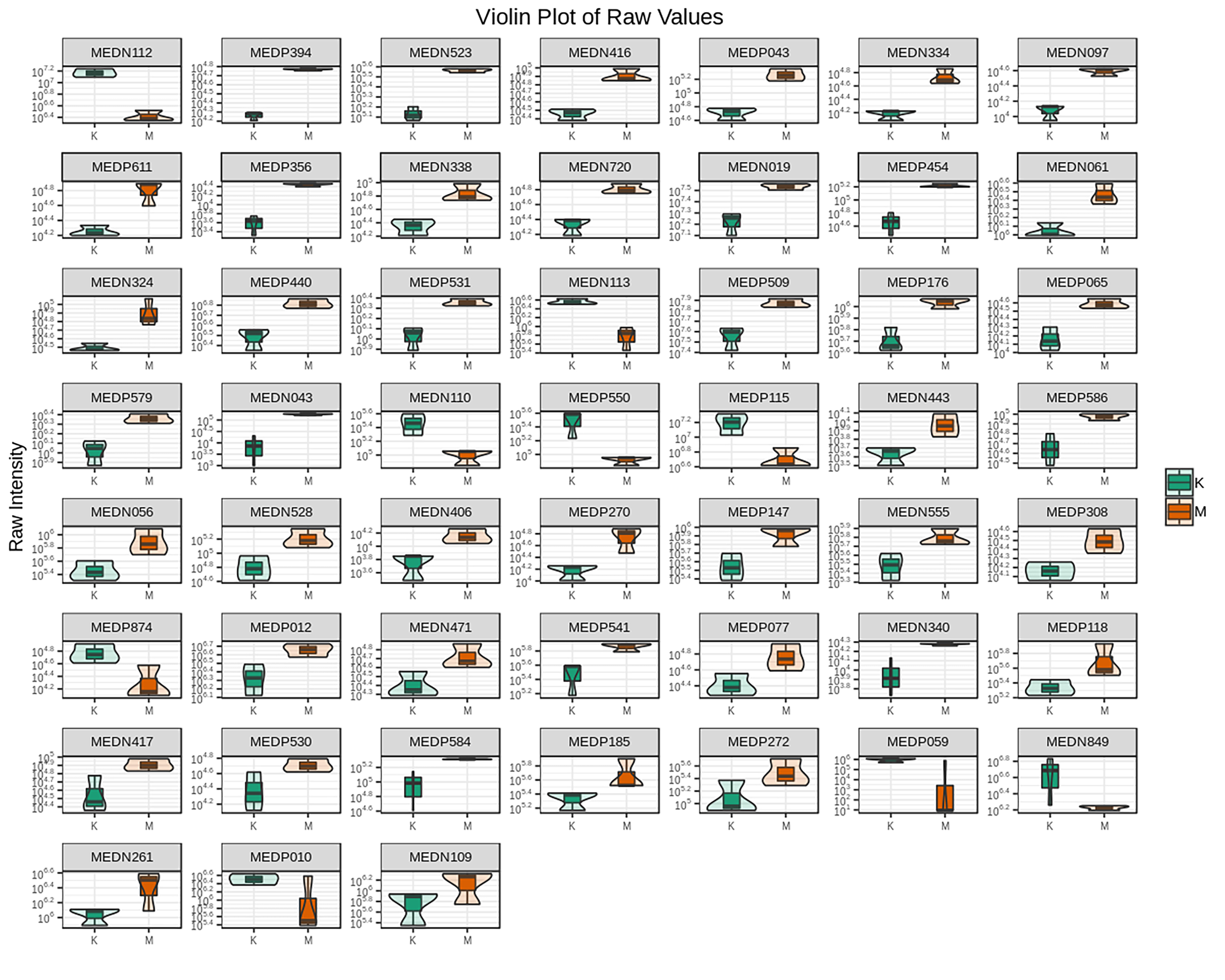

Supplement: Supplementary file 1 [file pharmaceuticals-18-00610-s001.zip › S10.jpg]

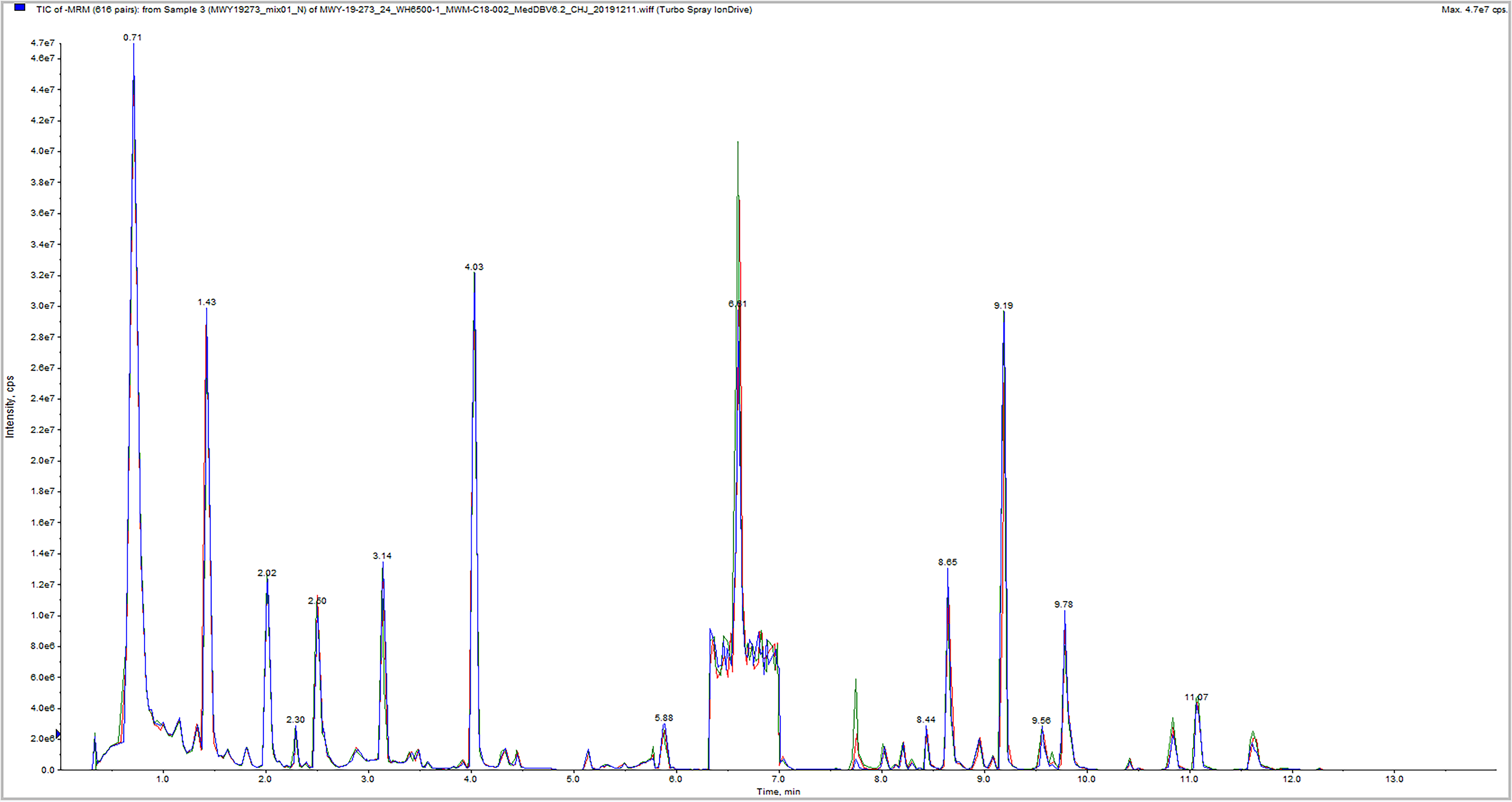

Supplement: Supplementary file 1 [file pharmaceuticals-18-00610-s001.zip › S2-1.jpg]

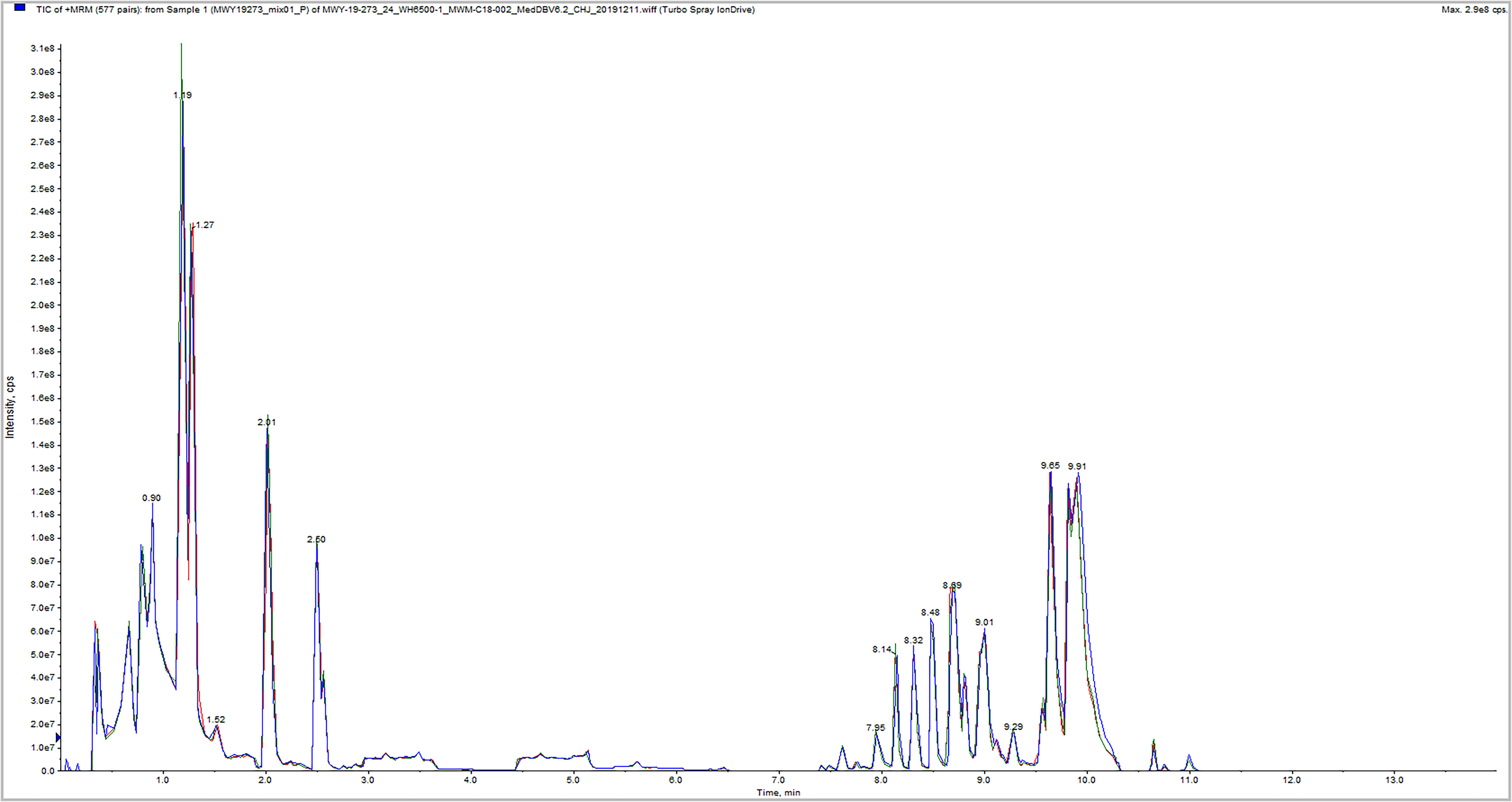

Supplement: Supplementary file 1 [file pharmaceuticals-18-00610-s001.zip › S2-2.jpg]

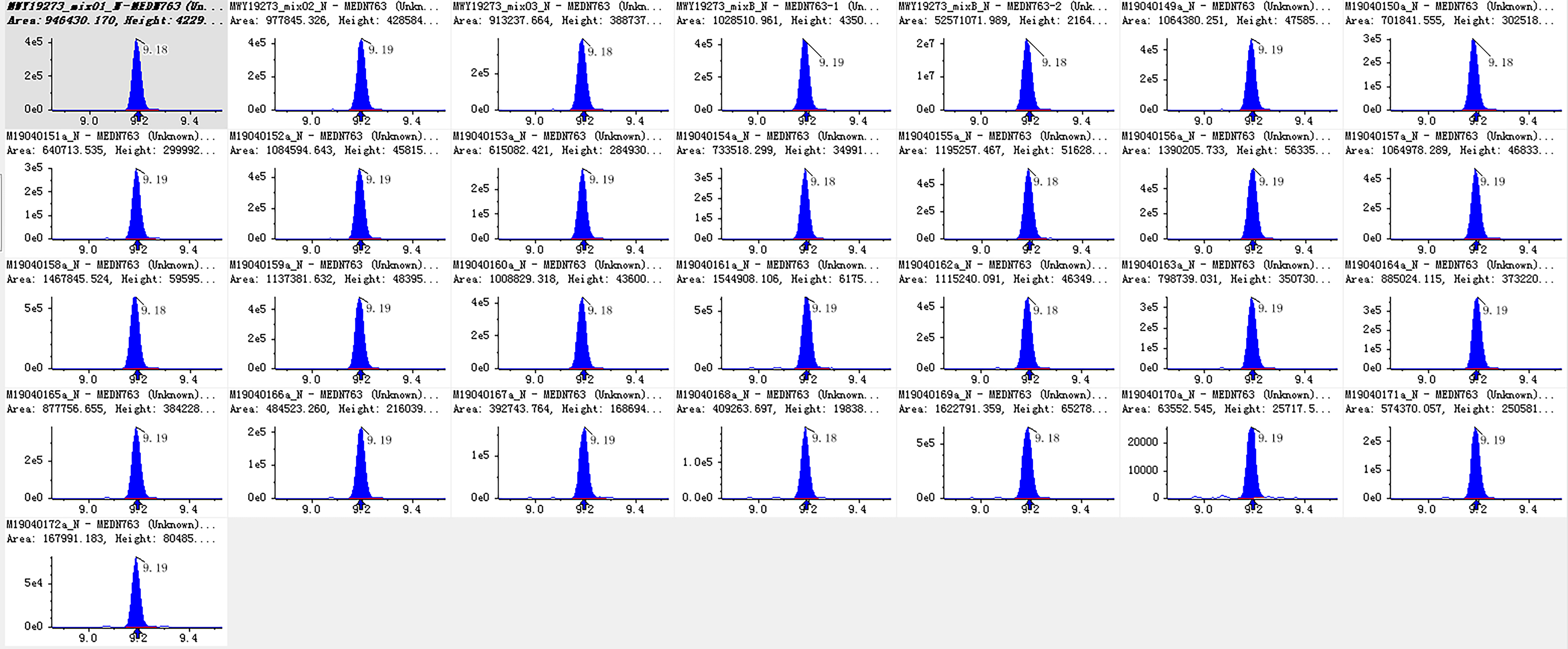

Supplement: Supplementary file 1 [file pharmaceuticals-18-00610-s001.zip › S3-1.jpg]

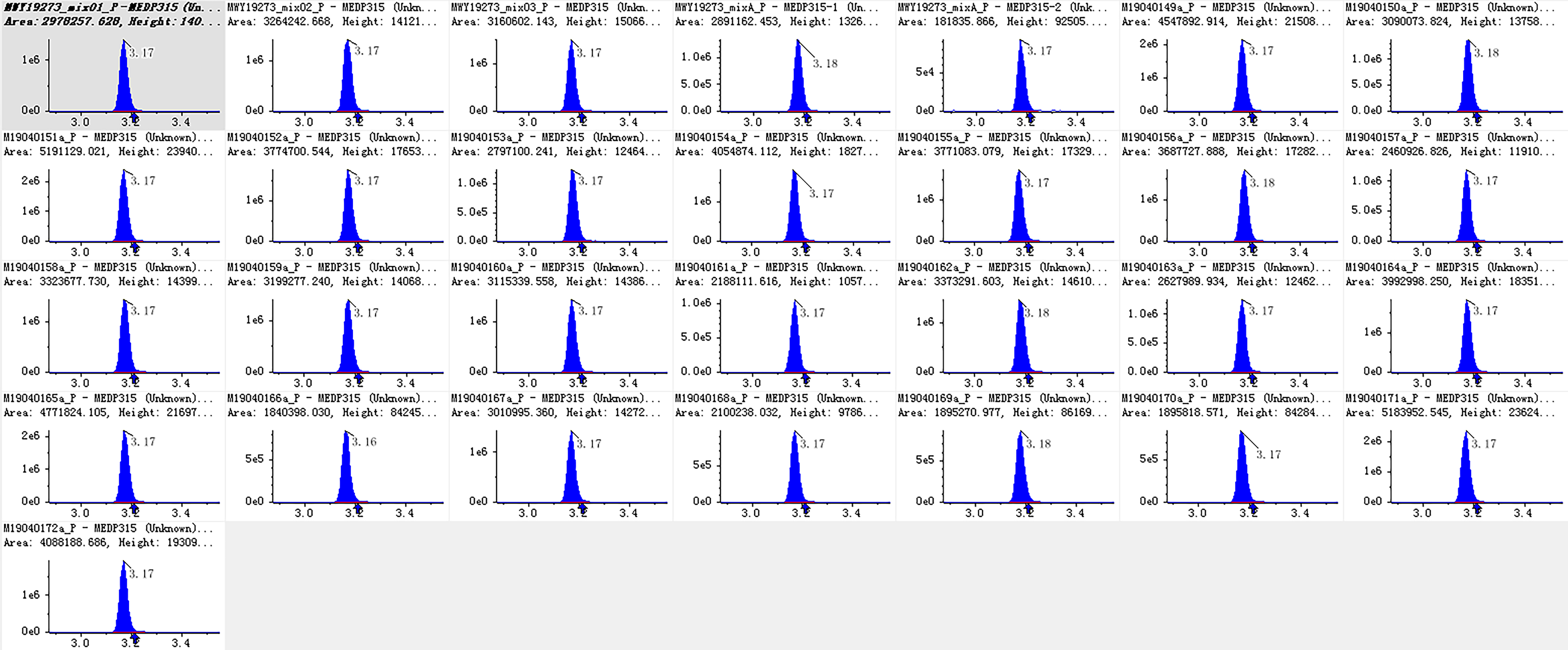

Supplement: Supplementary file 1 [file pharmaceuticals-18-00610-s001.zip › S3-2.jpg]

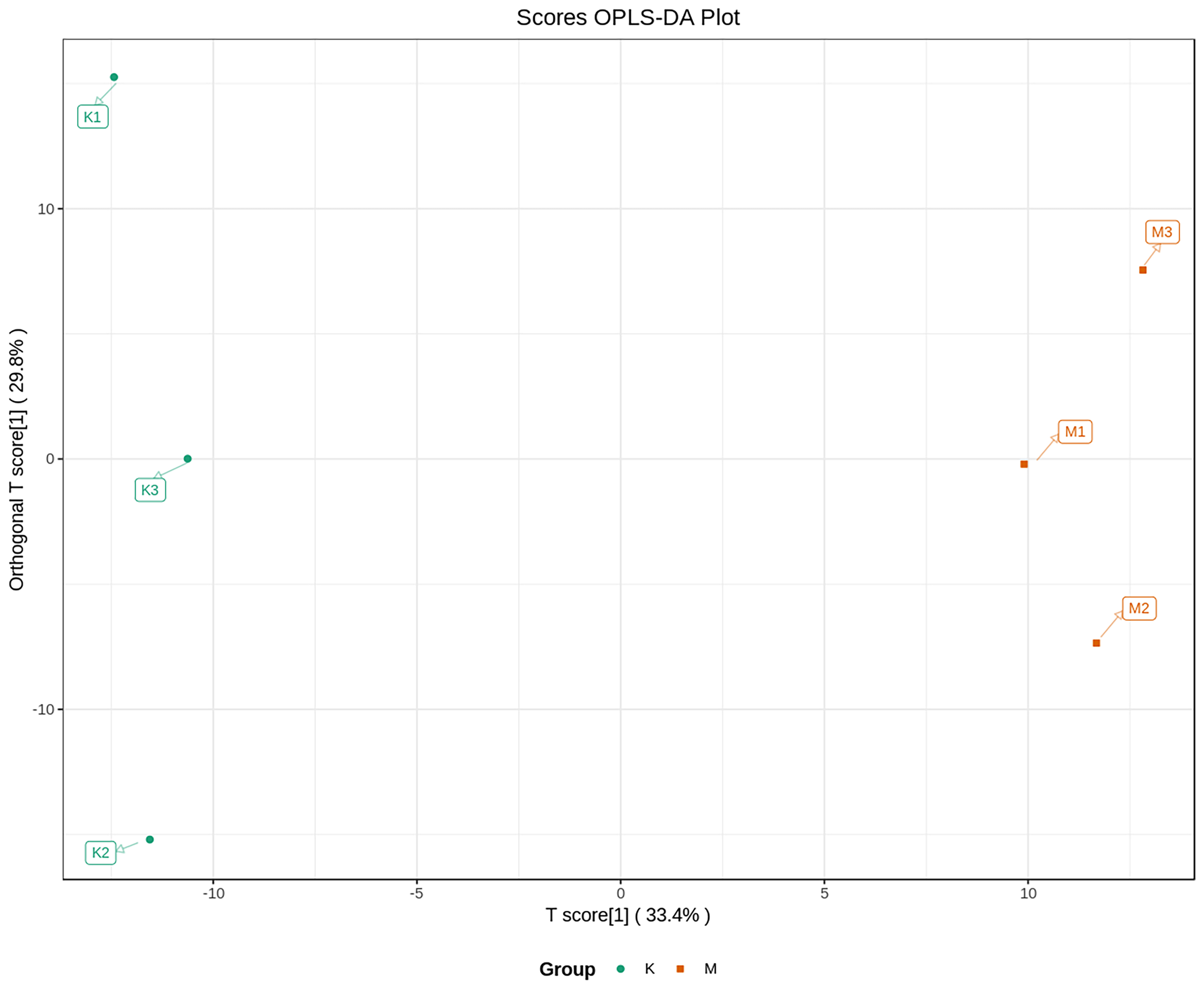

Supplement: Supplementary file 1 [file pharmaceuticals-18-00610-s001.zip › S4.jpg]

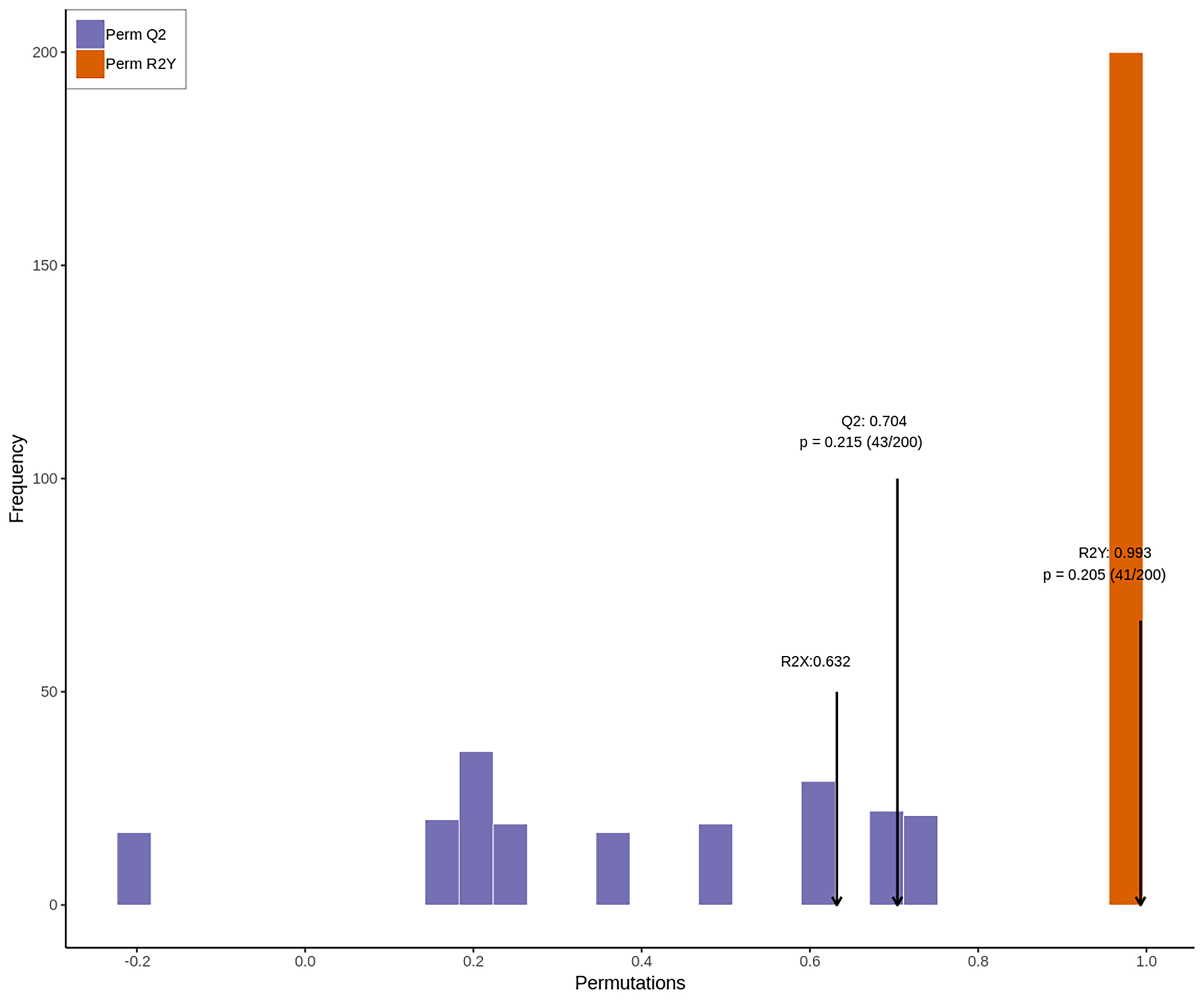

Supplement: Supplementary file 1 [file pharmaceuticals-18-00610-s001.zip › S5.jpg]

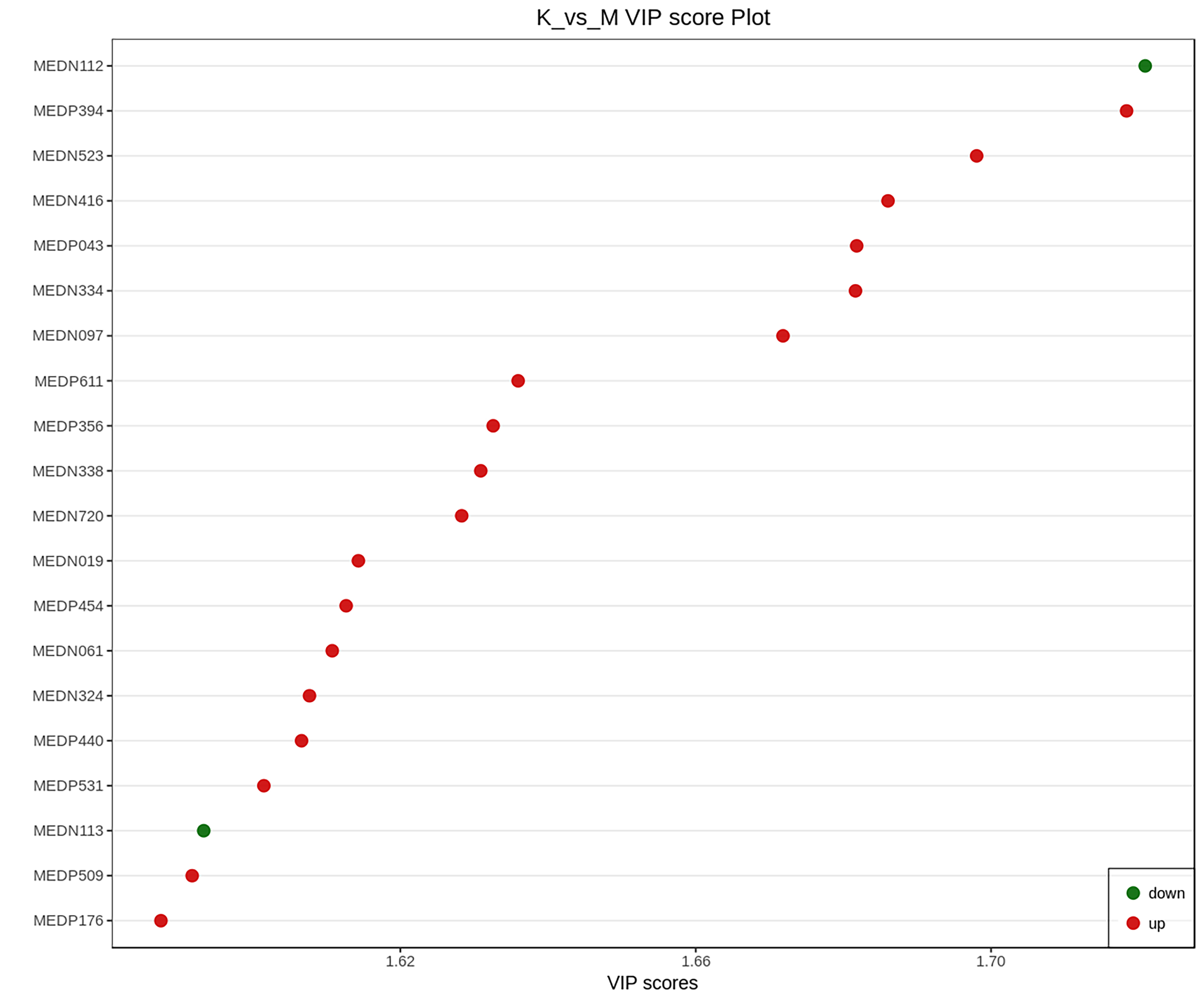

Supplement: Supplementary file 1 [file pharmaceuticals-18-00610-s001.zip › S6.jpg]

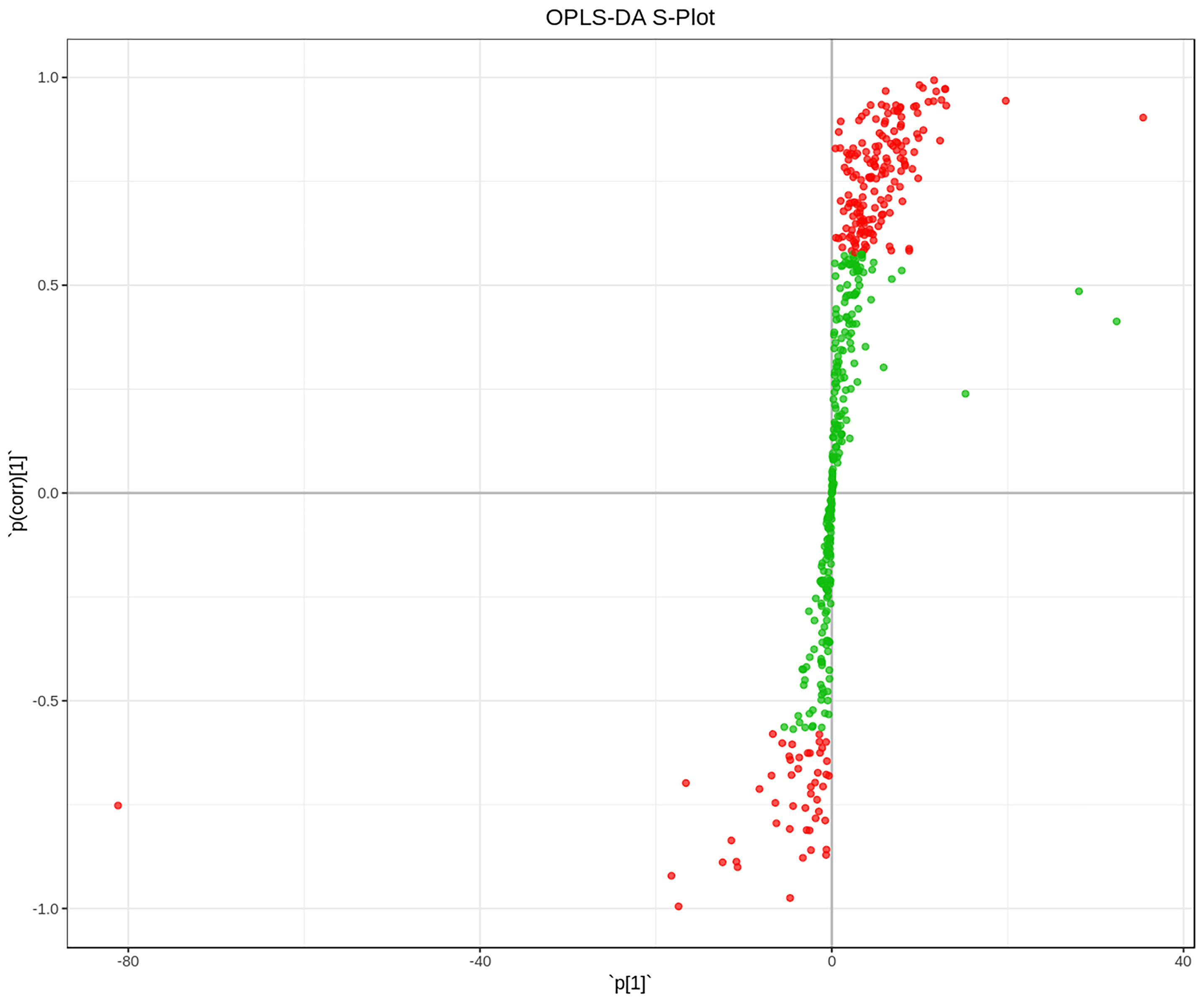

Supplement: Supplementary file 1 [file pharmaceuticals-18-00610-s001.zip › S7.jpg]

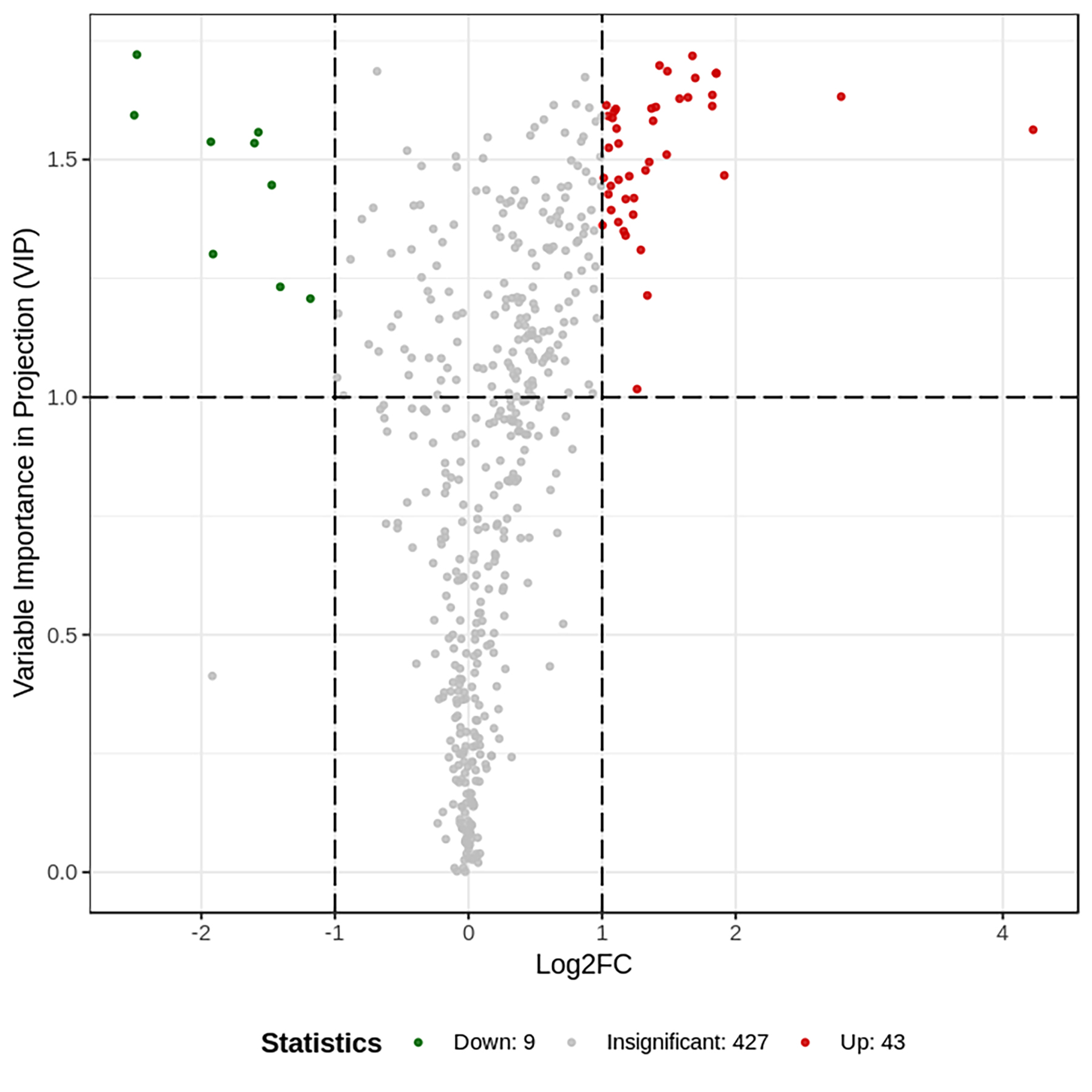

Supplement: Supplementary file 1 [file pharmaceuticals-18-00610-s001.zip › S8.jpg]

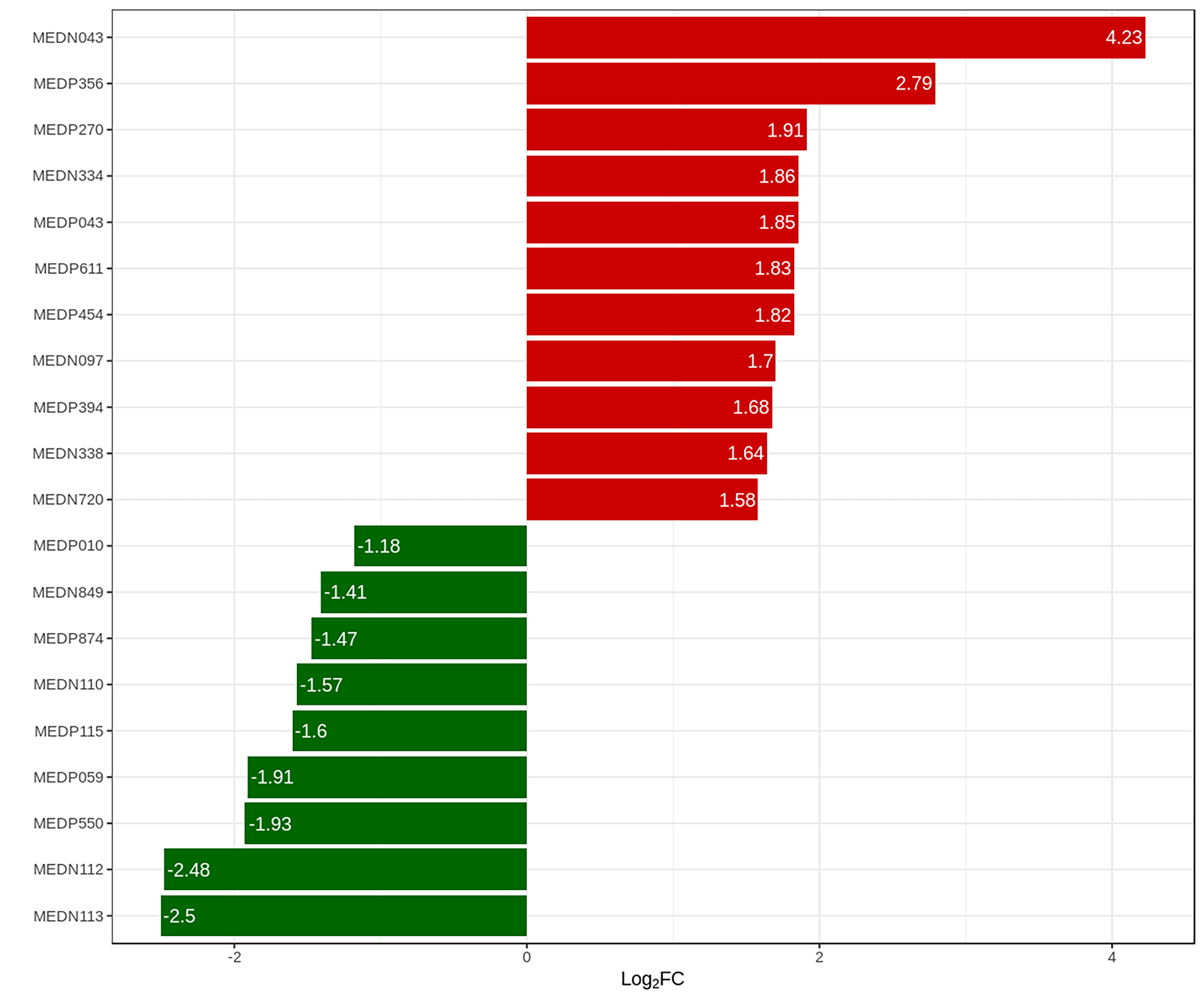

Supplement: Supplementary file 1 [file pharmaceuticals-18-00610-s001.zip › S9.jpg]
